# A personalized booklet for progressive foot and ankle exercises: a tool for continuous care of diabetic patients

**DOI:** 10.1186/1758-5996-7-S1-A237

**Published:** 2015-11-11

**Authors:** Jady Luara Verissimo, Isabel de Camargo Neves Sacco, Maria Helena Morgani de Almeida, Cristina Dallemole Sartor

**Affiliations:** 1 Universidade de Sao Paulo/Universidade Federal de Sao Paulo, São Paulo, Brazil

## Background

Diabetes mellitus causes great impact on foot and ankle function and mobility, mainly due to polyneuropathy. A continuous functional and musculoskeletal foot care is necessary to avoid complications, ideally with individualized intervention to promote less supervised activities.

## Objective

To develop a booklet that personalizes a foot and ankle routine of exercises according to the patients' individual improvements, to be used by patient with diabetes.

## Materials and methods

The development followed the Delphi method: (a) Production of the 1st version of the booklet: selection of information about self-assessment and foot care; creation of a male and female characters to dialogue with patients; selection of 7 simple and efficient exercises to increase foot strength and mobility; description of the exercises by pictures; and development of a table to control the progression of each exercise. A progression of approximately 6 levels of difficulty is available for each exercise that also differs in number of series and repetitions, body positions and materials used. After performing each exercise, patients have to classify their effort (easy, difficult, very difficult) and based on that, a progression will be made or not; (b) Professionals specialists' assessment: a jury was composed by 8 experts in dealing with diabetes. They assessed in the booklet the adequacy of the language, quantity and quality of the information and pictures provided, effectiveness of the selected exercises, motivation of daily practicing as a lifestyle, and contribution to be used as a tool to facilitate the communication between patients and health care providers.

## Results

Most specialists agreed (45%) or totally agreed (37%) with the content of the booklet but some changes were made according to their suggestions: (a) Inclusion of a visual effort face scale; (b) Adjust the table for controlling the exercises progression to be easier for the users; (c) Include a section to instruct health professionals about the benefits and usefulness of the booklet in a clinical setting.

## Conclusion

The booklet has valuable information about diabetes complications, and can promote a personalized progress of foot and ankle exercises routine. It stimulates the adherence of a daily practice and contributes to the continuous care. A broad disclosure of the booklet would give the population practical and useful information about the disease and have the potential to promote individualized health care.

